# The Intersection between Oral Microbiota, Host Gene Methylation and Patient Outcomes in Head and Neck Squamous Cell Carcinoma

**DOI:** 10.3390/cancers12113425

**Published:** 2020-11-18

**Authors:** Zigui Chen, Po Yee Wong, Cherrie W. K. Ng, Linlin Lan, Sherwood Fung, Jing W. Li, Liuyang Cai, Pu Lei, Qianqian Mou, Sunny H. Wong, William K. K. Wu, Ryan J. Li, Katie Meehan, Vivian W. Y. Lui, Chit Chow, Kwok W. Lo, Amy B. W. Chan, Siaw Shi Boon, Eric H. L. Lau, Zenon Yeung, Kwan C. Allen Chan, Eddy W. Y. Wong, Alfred S. L. Cheng, Jun Yu, Paul K. S. Chan, Jason Y. K. Chan

**Affiliations:** 1 Centre for Gut Microbiota Research, Faculty of Medicine, The Chinese University of Hong Kong, Hong Kong SAR, China; wonghei@cuhk.edu.hk (S.H.W.); wukakei@cuhk.edu.hk (W.K.K.W.); junyu@cuhk.edu.hk (J.Y.); paulkschan@cuhk.edu.hk (P.K.S.C.); 2 Department of Microbiology, The Chinese University of Hong Kong, Hong Kong SAR, China; bowieyee@pathology.hku.hk (P.Y.W.); liuyang.cai@link.cuhk.edu.hk (L.C.); leipu@link.cuhk.edu.hk (P.L.); qianqianmou@cuhk.edu.hk (Q.M.); boonss@cuhk.edu.hk (S.S.B.); 3 Li Ka Shing Institute of Health Sciences, The Chinese University of Hong Kong, Hong Kong SAR, China; sherwoodfyh@link.cuhk.edu.hk (S.F.); allen@cuhk.edu.hk (K.C.A.C.); 4 Department of Otorhinolaryngology, Head and Neck Surgery, The Chinese University of Hong Kong, Hong Kong SAR, China; cherrieng@ent.cuhk.edu.hk (C.W.K.N.); linlinlan@ent.cuhk.edu.hk (L.L.); marcoli@link.cuhk.edu.hk (J.W.L.); katiemeehan@ent.cuhk.edu.hk (K.M.); ericlau@ent.cuhk.edu.hk (E.H.L.L.); b128881@cuhk.edu.hk (Z.Y.); eddywywong@ent.cuhk.edu.hk (E.W.Y.W.); 5 Department of Chemical Pathology, The Chinese University of Hong Kong, Hong Kong SAR, China; 6 State Key Laboratory of Translational Oncology, Sir Y.K. Pao Centre for Cancer, The Chinese University of Hong Kong, Shatin, Hong Kong SAR, China; 7 Department of Medicine and Therapeutics, The Chinese University of Hong Kong, Hong Kong SAR, China; 8 Institute of Digestive Disease, State Key Laboratory of Digestive Disease, The Chinese University of Hong Kong, Hong Kong SAR, China; 9 CUHK Shenzhen Research Institute, Shenzhen, China; 10 Department of Anaesthesia and Intensive Care, The Chinese University of Hong Kong, Hong Kong SAR, China; 11 Department of Otolaryngology, Head and Neck Surgery, Oregon Health and Science University, Portland, OR 97239, USA; lry@ohsu.edu; 12 School of Biomedical Sciences, Faculty of Medicine, The Chinese University of Hong Kong, Hong Kong SAR, China; vlui002@cuhk.edu.hk (V.W.Y.L.); alfredcheng@cuhk.edu.hk (A.S.L.C.); 13 Department of Anatomical and Cellular Pathology, Faculty of Medicine, The Chinese University of Hong Kong, Hong Kong SAR, China; chit@cuhk.edu.hk (C.C.); kwlo@cuhk.edu.hk (K.W.L.); abwchan@cuhk.edu.hk (A.B.W.C.)

**Keywords:** HNSCC, microbiome, *Fusobacterium*, methylation, host-microbiome interaction

## Abstract

**Simple Summary:**

Recently, there has been increased recognition of an association between the bacterial microbiome and cancer. In this study, we characterized the non-HPV head and neck squamous cell carcinoma (HNSCC) microbiome. We found a significant enrichment of *Fusobacterium*, depletion of *Streptococcus*, and the microbial signatures of twelve bacterial genera distinguishing HNSCC. With increased *Fusobacterium*—in particular, *F. nucleatum*—in our HNSCC cohort and its known association with prognosis in colorectal cancers (CRC), we sought to further characterize the association between clinical outcomes and *F. nucleatum*, and the host interaction with *F. nucleatum*. We identified a higher abundance of *F. nucleatum* in non-smokers and an improved survival, in contrast to CRC. An integrative analysis also identified that the enrichment of *F. nucleatum* was associated with host gene promoter methylation, suggesting that the bacterial mircobiome status may have a potential role as a prognostic biomarker and be involved in the pathogenesis of HNSCC.

**Abstract:**

The role of oral microbiota in head and neck squamous cell carcinoma (HNSCC) is poorly understood. Here we sought to evaluate the association of the bacterial microbiome with host gene methylation and patient outcomes, and to explore its potential as a biomarker for early detection or intervention. Here we performed 16S rRNA gene amplicon sequencing in sixty-eight HNSCC patients across both tissue and oral rinse samples to identify oral bacteria with differential abundance between HNSCC and controls. A subset of thirty-one pairs of HNSCC tumor tissues and the adjacent normal tissues were characterized for host gene methylation profile using bisulfite capture sequencing. We observed significant enrichments of *Fusobacterium* and *Peptostreptococcus* in HNSCC tumor tissues when compared to the adjacent normal tissues, and in HNSCC oral rinses when compared to healthy subjects, while ten other bacterial genera were largely depleted. These HNSCC-related bacteria were discriminative for HNSCC and controls with area under the receiver operating curves (AUCs) of 0.84 and 0.86 in tissue and oral rinse samples, respectively. Moreover, *Fusobacterium nucleatum* abundance in HNSCC cases was strongly associated with non-smokers, lower tumor stage, lower rate of recurrence, and improved disease-specific survival. An integrative analysis identified that enrichment of *F. nucleatum* was associated with host gene promoter methylation, including hypermethylation of tumor suppressor genes *LXN* and *SMARCA2*, for which gene expressions were downregulated in the HNSCC cohort from The Cancer Genome Atlas. In conclusion, we identified a taxonomically defined microbial consortium associated with HNSCC that may have clinical potential regarding biomarkers for early detection or intervention. Host–microbe interactions between *F. nucleatum* enrichment and clinical outcomes or host gene methylation imply a potential role of *F. nucleatum* as a pro-inflammatory driver in initiating HNSCC without traditional risk factors, which warrants further investigation for the underlying mechanisms.

## 1. Background

Known etiological factors involved in the development of head and neck squamous cell carcinoma (HNSCC) include smoking, alcohol abuse, and areca nut chewing [1,2]. However, the prevalence of HNSCC without traditional risk factors has been increasing [3]. Human papillomavirus (HPV) plays an etiological role in a proportion of oropharyngeal squamous cell carcinoma but has no clear association with SCC of other sites in the head and neck region [3]. Emerging data implicate the involvement of the human bacterial microbiome in a variety of cancers, most notably *Fusobacterium* in colorectal cancer [4,5] and *Helicobacter* in gastric cancer [6,7]. The bacterial microbiome has also been proposed to interact with the host and play a role in carcinogenesis through host epigenetic alterations in the gut mucosa that are seen in ulcerative colitis and colorectal cancer [8,9,10].

In the head and neck region, the oral bacterial microbiome has been implicated in the development of dental caries with acidophilic bacteria that result in the demineralization of enamel and resultant caries [11]. Other poor oral health conditions, including chronic periodontitis and gingivitis [12], have also been implicated in HNSCC, as it may result in a change in the oral microbiome leading to chronic inflammation and cancer progression [13,14]. Recently, there have been studies reporting alterations in the microbiome of HNSCC [15,16,17,18]. A significant loss in microbial diversity, and changes in the relative abundance of some oral bacteria, such as *Fusobacteria* and *Streptococcus*, have been observed in HNSCC patients. However, many of these studies had limitations in sampling methods, did not control for HPV status and did not evaluate the host–microbiome interactions [17,18,19,20].

Here we sought to characterize the non-HPV HNSCC microbiome using both tissues and oral saliva rinses, in order to delineate the microbial consortium associated with HNSCC. Microbiota of HNSCC tumor tissues and the adjacent normal tissues were compared, and oral rinse samples from the same HNSCC patients were collected to compare them with those from matched healthy subjects. Finally, with these data we correlated the bacterial microbiome with host genome methylation pattern and patient outcomes.

## 2. Methods

### 2.1. Ethical Approval

This study has been approved by the approved by The Joint Chinese University of Hong Kong–New Territories East Cluster Clinical Research Ethics Committee (CREC reference number 2015.396, 2017.143). Patients with head and neck squamous cell carcinoma who were admitted into the Prince of Wales Hospital and United Christian Hospital in Hong Kong and agreed with written informed consent were recruited between October 2015 and April 2018. All cases were reviewed by a pathologist. Separately, age, gender, and smoking-matched healthy individuals above 18 years of age with no history of malignancies were recruited from Prince of Wales Hospital.

### 2.2. HNSCC Tissue Collection and Processing

Tumor tissues and the adjacent normal (AN) tissues were collected at the time of surgery. Briefly, around 5 mm^3^ tumor samples were excised from the tumor mass without involving the margin. Paired normal tissues ≥ 5 cm away from the margin of the tumor were excised. These specimens were copiously irrigated with sterile saline to wash away surface contamination prior to collection. Tissue specimens were stored at −80 °C until further use.

### 2.3. Collection of Oral Rinse Samples

Oral rinse samples of HNSCC patients were collected in the hospital prior to surgery before any intraoperative antibiotics. In brief, pre-treatment 30 mL oral rinses were obtained with normal saline gargled twice, for 20 and 10 s respectively, as previously described [21,22]. Oral rinse samples from healthy subjects were collected using the same protocol. Oral rinses were sent to the laboratory within one hour, and were centrifuged at 1600× *g* for 10 min in 4 °C immediately. Supernatants were discarded and the cellular pellet was stored at −80°C until further use.

### 2.4. DNA Extraction of Tissues and Oral Rinses

Fresh frozen tissue samples of around 10–20 mg were manually homogenized into small pieces and treated with 20 μL proteinase K at 55 °C overnight to lyse cell membranes. Disaggregated samples were extracted for total DNA using the Qiagen DNeasy Blood and Tissue Kit (Qiagen, Valencia, CA, USA) following the manufacturer’s protocol. Purified DNA was eluted into 100 μL elution buffer and stored in −20 °C. For oral rinsing, total DNA was extracted from pellets centrifuged from 1–2 mL of oral rinse solution using the QIAamp DNA Mini Kit (Qiagen, Valencia, CA, USA), and eluted into 100 μL elution buffer for further use.

### 2.5. HPV Genotyping

HPV genotyping was performed using two PCR-based amplicon sequencing assays targeting the conserved L1 open reading frame (ORF) of HPV [23]. A pair of dual 12 bp barcodes were introduced to the PCR amplicons using forward and reverse primers; PCR mixtures were sequenced on an Illumina MiSeq (Illumina, San Diego, CA, USA) at the Weill Cornell Medicine Genomics Resources Core Facility, New York, USA, using paired-end 150 bp reads. Following demultiplexing, short reads passing the quality filter were blastn-searched against a PV reference database using UPARSE software [24]. An operational taxonomic unit (OTU) count table was created using a 90% identity threshold assigning each OTU with a PV type.

### 2.6. 16S rRNA Gene PCR Amplification and Sequencing

Extracted DNA was used for oral microbiota profiling by sequencing the bacterial 16S rRNA gene V3-V4 region, with a primer set of 341F (5′−CCT ACG GGN GGC WGC AG−3′) and 806R (5′−GGA CTA CNV GGG TWT CTA AT−3′) plus minor modifications as previously described [25]. A pair of dual 12 bp barcodes was indexed to each amplicon set through the forward and reverse primers modified from the Earth Microbiome Project protocol [26]. Successful amplicons were equally pooled and sequenced on an Illumina MiSeq (Illumina, San Diego, CA, USA) at the Weill Cornell Medicine Genomics Resources Core Facility, New York, USA, using paired-end 300 bp reads. For quality control, each sequencing batch included a mock community, DNA negative controls, and technical replicate samples.

### 2.7. 16S Sequence Data Processing and Community Composition Statistical Analyses

Following short reads demultiplexing, the QIIME2 (v2019.7) package [27], including pipelines for quality control, dada2 denoising, and sequence clustering, was applied to process 16S rRNA short reads into an amplicon sequence variant (ASV) count table. The SILVA v132 99% 16S rRNA gene reference database was used to assign taxonomic identities to ASVs at genus and phylum levels, respectively. All reads assigned to mitochondria and chloroplasts were excluded. The *Fusobacterium* ASVs were further assigned at the species level using pplacer [28] by placing short reads on a phylogenetic tree inferred from the 16S rRNA gene complete sequences of *Fusobacterium* to maximize phylogenetic likelihood.

In order to retain all samples for diversity analysis, reads from each sample were rarefied to a depth of 2000 mean reads, after repeating for 100 times, to normalize the data for differences in sequencing depth among samples. Bacterial taxa with ≥1% relative abundance in at least one sample were retained. The diversity of observed bacterial genus taking into consideration species richness, and the effective numbers of Shannon and Simpson indexes, were calculated. Pairwise Bray–Curtis dissimilarities between samples were calculated using scripts in R v3.4.0 package. Differences in community composition were assessed using permutational multivariate analysis of variance (PERMANOVA) in the Vegan R package. Principal coordinate analysis was performed to visualize associations between community composition. Comparisons of the relative abundances of ASVs or bacterial taxa between defined groups were performed using nonparametric Mann–Whitney Wilcoxon rank sum test (MWU), Wilcoxon signed rank test (WSR), Kruskal–Wallis test (KW), or Tukey’s honest significant difference (Tukey HSD) post hoc test, where appropriate. Receiver operating characteristic (ROC) curve analyses, including calculation of area under the ROC curve (AUC), were used to evaluate the ability of bacterial genera to distinguish cases from controls. A two-sided *p* value of ≤0.05 and/or a false discovery rate (FDR)-adjusted *p* value (*q* value) of ≤0.05 was used as the threshold for significance.

### 2.8. Microbial Rank Correlation and Identifying Indicator Markers

Networks between bacterial genera were determined by calculating the pairwise SparCC correlation coefficient using microbial reads counts [29]. Two bacteria genera were considered statistically correlated if the absolute value was ≥0.3 and the *p* value was ≤0.05. Cytoscape v3.7.1 was used to visualize the network [30].

### 2.9. Functional Prediction Based on 16S rRNA Gene Community Composition

Functional profiles of microbial communities were predicted using PICRUSt2 (https://github.com/picrust/picrust2/wiki) based on 16S rRNA gene sequences represented as Kyoto Encyclopedia of Genes and Genomes (KEGG) Orthology (KO) and metabolic MetaCyc pathway counts [31]. Nine samples with nearest sequenced taxon index (NSTI) values ≥0.15 were excluded due to low accuracy of the functional prediction. Differential abundances of KO pathways for the comparison between conditions were analyzed using the DESeq2 R package [32].

### 2.10. Fusobacterium nucleatum in Situ Hybridization (ISH) Analysis

Six primary HNSCC cases with *F. nucleatum* on qPCR in fresh tissue had archival formalin-fixed paraffin-embedded (FFPE) samples retrieved to determine the spatial distribution of Fusobacterium in HNSCC tissues. Briefly, 4 um-thick FFPE sections were subjected to RNA in situ hybridization with B-FNucleatum-fadA RNA probe and RNAscope^®^ 2.5 HD Reagent Kit-BROWN according to the protocol suggested by the manufacturer (ACD). Negative control probe—DapB (catalogue number 310043), and positive control probe—Hs-PPIB (catalogue number 313901), were used for negative and RNA quality controls respectively. The slides were evaluated at high magnification (40×) fields per sample by a dedicated head and neck pathologist. The digital microscope camera Nikon DS-Ri2 digital microscope camera and software NIS Elements Imaging Software Version 4.60 were used to capture the images.

### 2.11. Quantitative PCR (qPCR) for Fusobacterium nucleatum

A TaqMan primer/probe set targeting the *nusG* gene was used to amplify *Fusobacterium nucleatum* DNA as previously described [33]. The fold difference (2^-ΔΔCt^) in *F. nucleatum* abundance in tumor versus AN tissue was calculated by subtracting ΔCt_tumor_ from ΔCt_AN_, where ΔCt is the difference in threshold cycle number for the *nusG* and PGT assay. PCR amplification was performed using Taqman Universal Master Mix, with primer and probe sequences as follows: *Fusobacterium nugG* forward primer, 5′ AAC CAT TAC TTT AAC TCT ACC ATG TTC A 3′; *nugG* reverse primer, 5′ TTG ACT TTA CAG AAG GAG ATT ATG TAA AAA TC 3′; *nugG* FAM probe, 5′ TCA GCA ACT TGT CCT TCT TGA TCT TTA AAT GAA CC 3′; PGT forward primer, 5′ ATC CCC AAA GCA CCT GGT TT 3′; PGT reverse primer, 5′ AGA GGC CAA GAT AGT CCT GGT AA 3′; PGT FAM probe, 5′ CCA TCC ATG TCC TCA TCT C 3′.

### 2.12. Human DNA CpG Methylation from HNSCC Tissue

Total DNA from HNSCC tumor and AN tissue was pre-prepared for illumine library using KAPA HTP Library Preparation Kit (Kapa Biosystems, Wilmington, MA, USA) and then exposed to sodium bisulfite at 60 °C in the dark to convert unmethylated cytosine nucleotides into uracil nucleotides using EpiTect DNA Bisulfite Kit (Qiagen, Valencia, CA, USA). The SeqCap Epi CpGiant System interrogating more than 5.5 million CpG sites (Roche, USA) was used to probe bisulfite-converted human DNA libraries for Illumina HiSeq sequencing (Illumina, San Diego, CA, USA) at NovoGene, China, using paired-end 150 bp reads.

Short reads were quality trimmed using Trimmomatic [34] and mapped to the human genome (hg38) using Bismark [35]. Mapped bam files were sorted, and duplicated reads were removed. Methylation calls from bam files were further imported into methylKit [36] to call differentially methylated regions (DMRs) of 1000 bp size genome-wide. Promoters were defined by taking 3000 bp up- and down-stream of the transcription start site. Age, gender, smoking status, drinking status, and *Fusobacterium* abundance (Fuso-high vs. Fuso-low) for each individual were included in the model as confounding factors. Five groups of comparison were conducted, including (1) tumor vs. adjacent normal tissues, (2) *Fusobacterium*-high vs. *Fusobacterium*-low in tumor tissues, (3) *Fusobacterium*-high vs. *Fusobacterium*-low in adjacent normal tissues, (4) tumor vs. adjacent normal tissues in *Fusobacterium*-high group, and (5) tumor vs. adjacent normal tissues in *Fusobacterium*-low group. The methylation status was determined in percentage based on the actual C bases in the reads aligning to a given cytosine location in DMR. Hyper- and hypo-methylated regions were identified if the methylation difference were ≥15% (adjusted *p* ≤ 0.01), as measured by *calculateDiffMeth* in methylKit. The association between *Fusobacterium* enrichment and gene promoter methylation was regarded as significant if the tests of comparisons 1, 2, and 4 but not 3 and 5 met criteria, which were further repeated by a negative binomial generalized linear regression model test and a Spearman’s rank-order correlation test, complied with *glm.nb* and *cor.test*, respectively (*p* ≤ 0.05). A volcano plot was used to visualize the DMRs.

To explore the association of DNA methylation in the promoter region with gene transcription, The Cancer Genome Atlas (TCGA) HNSCC cohort RNA-seq raw counts datasets composed of 500 tumor and 38 control tissue samples were retrieved using R package curatedTCGAData. Differential gene expression profiling was measured using DESeq2 using a cutoff of log2 fold change (log2FC) ≥1 and adjusted *p* ≤ 0.01. The KEGG 2019 human database at Enrichr (https://amp.pharm.mssm.edu/Enrichr/) was accessed to predict gene pathways.

## 3. Results

### 3.1. Study Subjects

Sixty-eight HNSCC patients, after excluding 13 high-risk (HR) HPV-positive cases, were included in this study, from which samples including oral rinses, tumor tissues, and the adjacent normal (AN) tissue were collected. Oral rinses from sixty-eight age and gender matched non-HNSCC subjects were collected as controls. There were no significant differences between HNSCC patients and control subjects in age, gender, smoking, and alcohol history (Table S1). All samples were negative for high-risk HPV DNA by PCR.

### 3.2. Differential Oral Microbial Community in HNSCCs Compared to Controls

**Figure 1:**
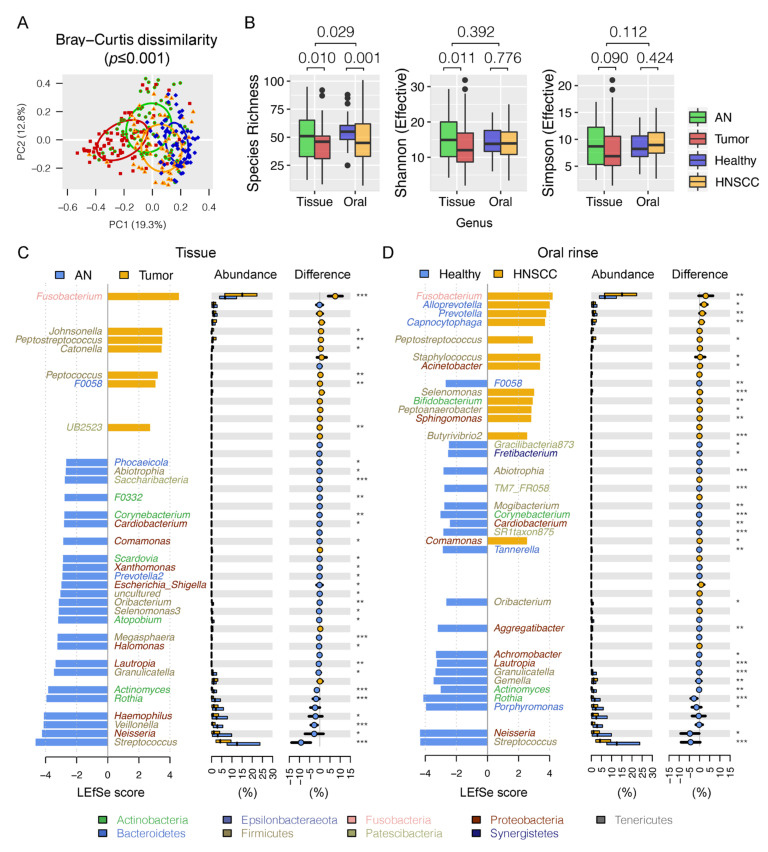
Oral microbiota dysbiosis associated with head and neck squamous cell carcinomas (HNSCCs). (**A**) Principal coordinates plot using Bray–Curtis dissimilarity. (**B**) Comparison of oral microbiota diversity summarized at the genus level. (**C**) Comparison of oral microbiota between HNSCC tumor (tumor) and adjacent normal (AN) tissues, with bacterial genera that best discriminated HNSCCs and controls by a linear discriminant analysis (LDA) for effect size (LEfSe). The bar length represents log10 LDA score. Difference in the relative abundance tested by pairwise Tukey HSD post hoc is shown on the right panel. (**D**) Comparison of oral microbiota between HNSCC oral rinses and healthy subjects. Adjusted Mann–Whitney *U*-test *p* value is marked with * if ≤ 0.05, ** ≤ 0.01, or *** ≤ 0.001.

Overall, Firmicutes (relative abundance of 30.0 ± 1.0%), Proteobacteria (24.4 ± 1.2%), and Bacteroidetes (21.5 ± 0.7%) were the three most predominant bacterial phyla in the surveyed samples. When bacterial taxa were summarized at genus level, a permutational multivariate analysis of variance (PERMANOVA) using Bray–Curtis distances indicated that approximately 12.9% of variation in microbial composition could be attributed to the oral habitats (tissue vs. oral rinse) (df = 1, R^2^ = 0.086, pseudo-F = 26.483, *p* ≤ 0.001), disease status (HNSCCs vs. controls) (df = 1, R^2^ = 0.037, pseudo-F = 11.290, *p* ≤ 0.001), and the combination (df = 1, R^2^ = 0.006, pseudo-F = 1.887, *p* = 0.029), wherein oral habitat was the main source of variability, as supported by a principal coordinate analysis (PCoA) (Figure 1A). Decreased alpha diversity of oral microbiota was observed in tumor tissues when compared to the adjacent normal tissues, as measured by taxa richness and the effective numbers of Shannon index (Figure 1B). However, no significant differences in the effective numbers of Shannon or Simpson diversities were observed between oral rinses collected from HNSCC and healthy subjects.

**Figure 2:**
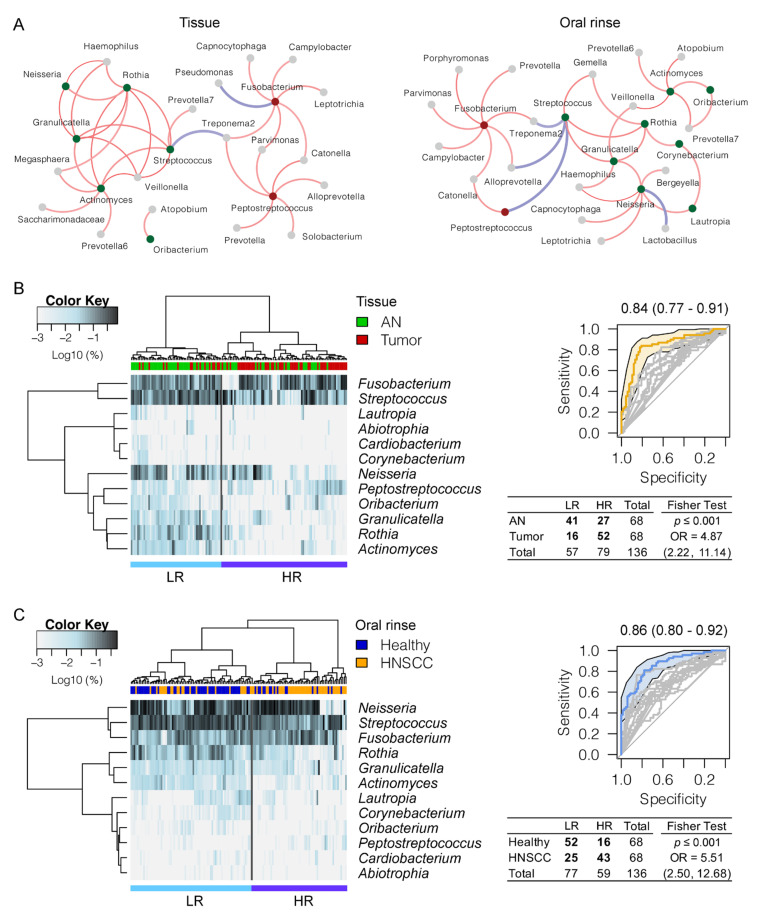
A refinement of twelve “core” bacterial genera discriminating HNSCCs from controls. (**A**) A SparCC correlation analysis (*r* ≥ 0.3, *p*≤ 0.01) showing connections between pathogen–pathogen, nonpathogen–nonpathogen, and pathogen–nonpathogen bacteria. Grey dots indicate bacterial genera with no significant differences in abundance between HNSCC and controls. Red and green dots indicate bacterial genera enriched in HNSCCs and controls, respectively. Connections with red and blue lines indicate collaborative and inhibitive relationships, respectively. Hierarchical cluster analyses of tissues (**B**) and oral rinses (**C**) using distance matrix of 12 discriminative bacterial genera classified oral microbial communities into two clades, namely, high-risk and low-risk, based on the dendrogram topologies. The receiver operating characteristic (ROC) analysis of combined twelve genera achieved AUCs of 0.84 and 0.86 in tissues and oral rinses, respectively. Clustering between the two groups was evaluated using a two-tailed Fisher exact test.

Using the linear discriminant analysis effect size (LEfSe) algorithm, we were able to distinguish oral bacteria between HNSCCs and controls. In total, there were seven significantly enriched and 25 depleted bacterial genera in HNSCC tumor tissues when compared to the adjacent normal tissues (Figure 1C, Table S2). Similarly, we found 13 bacterial genera largely dominated in abundance in HNSCC oral rinses, and 21 in healthy subjects (Figure 1D). These bacteria may represent pathologic or commensal microbiota in the upper aerodigestive tract. For example, *Fusobacterium*, the most predominant periodontal pathogen observed in 90% of the surveyed samples (≥1% relative abundance), was nearly double in relative abundance in HNSCC when compared to controls (tissue: 16.3% vs. 8.6%, *p* ≤ 0.001; oral rinse: 7.7% vs. 4.6%, *p* ≤ 0.001). In contrast, a dramatic reduction in relative abundance of the commensal *Streptococcus* in HNSCC was observed (tissue: 8.0% vs. 17.2%, *p* ≤ 0.001; oral rinse: 10.6% vs. 15.0%, *p* ≤ 0.001). In particular, we found two bacterial genera (*Fusobacterium* and *Peptostreptococcus*) to be consistently increased in relative abundances both in tumor tissues and HNSCC oral rinses, whereas ten taxa (*Streptococcus*, *Neisseria, Rothia*, *Actinomyces*, *Granulicatella*, *Oribacterium*, *Lautropia*, *Corynebacterium*, *Abiotrophia*, and *Cardiobacterium*) were significantly decreased, with area under the receiver operating curve (AUC) values ranging between 0.58 and 0.80 (Figure S1, Table 1), although bacteria may manifest as different colonies in different ecological habitats (tissues vs. oral rinses) (Figure S2). Using SparCC correlation analysis (*r* ≥ 0.3, *p* ≤ 0.01), we explored the relationships among bacterial genera with consistent changes in abundance both in tissue and oral rinse samples, and found two main network clusters represented by periodontal pathogens (e.g., *Fusobacterium*, *Peptostreptococcus*) and commensal bacteria (e.g., *Streptococcus, Rothia*, *Granulicatella*) (Figure 2A). The HNSCC-related pathogenic genera (red and green dots) further formed pathogen–pathogen or nonpathogen–nonpathogen collaborative connections (red lines) with other non-pathogenic bacteria (grey dots), such as *Fusobacterium*–*Treponema2*, *Peptostreptococcus*–*Catonella*, *Neisseria*–*Haemophilus*, and *Granulicatella*–*Veillonella*. An inhibitive network of bacteria (blue lines) was not common within the surveyed samples and was mainly observed between a pathogen and a nonpathogen (e.g., *Streptococcus*–*Treponema2* and *Streptococcus*–Alloprevetolla).

### 3.3. Panel of the Bacterial Microbiome Useful in Differentiating HNSCC from Controls

To evaluate the potential use of oral microbiota for biomarkers in HNSCC, we performed hierarchical clustering to assign the surveyed samples into two groups, namely, high risk (HR) and low risk (LR), based on the relative abundance of twelve HNSCC-related bacterial genera with consistent changes both in tissue and oral rinse samples (Figure 2B,C). Sample clustering into the HR group comprised the majority of HNSCCs (76%, 52/68 of tumor tissues; 63%, 43/68 of HNSCC oral rinses), and the LR group was primarily controls (60%, 41/68 of adjacent normal tissues; 76%, 52/68 of healthy oral rinses), with odds ratios of 4.87 (*p* ≤ 0.001) and 5.51 (*p* ≤ 0.001) in tissues and oral rinses, respectively. A combination of these 12 bacterial genera achieved an AUC of 0.84 (95% CI 0.77–0.91) in tissues and 0.86 (95% CI 0.80–0.92) in oral rinses for distinguishing HNSCCs from controls, suggesting the potential utility of bacterial discrimination for biomarkers in HNSCC clinical management.

### 3.4. Pathways Related to Bacterial Adaptation and Infectious Disease Enriched in HNSCC

Tumor microenvironment may enrich for functionally similar microbial communities. We used Phylogenetic Investigation of Communities by Reconstruction of Unobserved States 2 (PICRUSt2) to predict Kyoto Encyclopaedia of Genes and Genomes (KEGG) Ortholog (KO) abundance profiles based on the composition of microbial communities. Differentially abundant and depleted pathways were observed between HNSCCs and controls (Table S3), including twenty functions with consistent changes both in tissue and oral rinse habitats (Figure S3). The most significant enrichments (*p* ≤ 0.05) involved the plant–pathogen interaction (ko04626) and epithelial cell signaling (ko05120), which were mainly contributed by *Fusobacterium* and *Leptotrichia* (Table S4). In contrast, depletions in abundance of *Rothia*, *Lautropia*, *Neisseria*, and several commensal bacteria were largely responsible for the suppression of pathways related to proteasome (ko03050), p53 signaling pathway (ko04115), and ribosome biogenesis in eukaryotes (ko03008). We also observed enriched pathways related to bacterial cell motility (ko02030: bacterial chemotaxis, ko02040: flagellar assembly) in tumor tissues, implying a favorable microenvironment of niche adaptation for periodontal pathogens such as *Treponema2*, *Fusobacterium*, and *Catonella*.

### 3.5. Fusobacterium nucleatum is Associated with HNSCC Patient Outcomes

**Figure 3:**
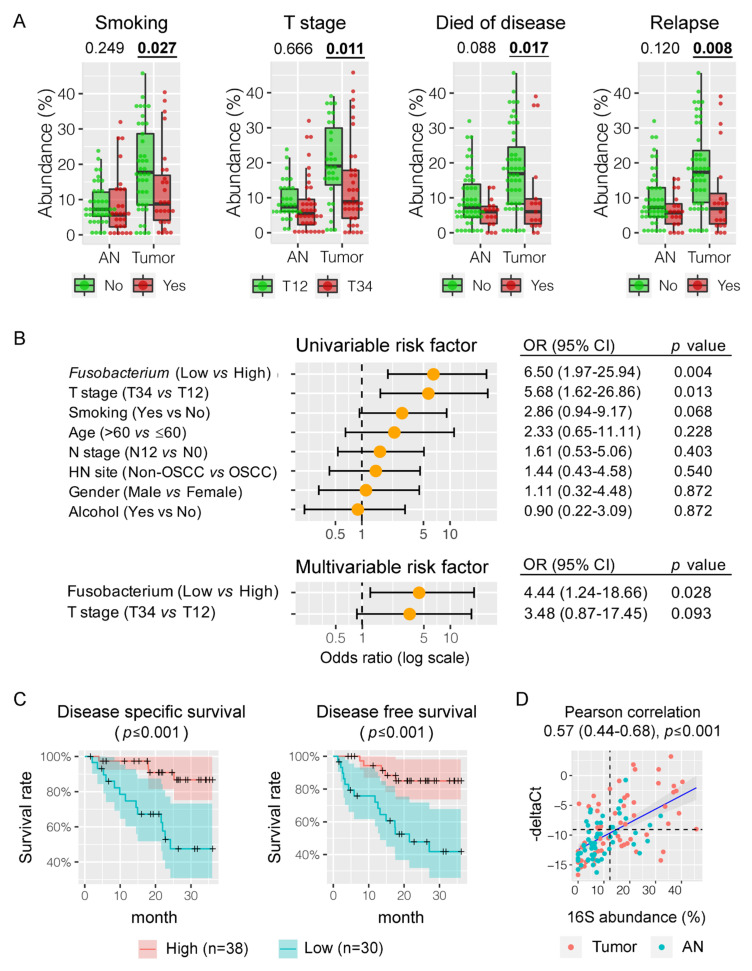
Association of *Fusobacterium* abundance with HNSCC patient outcomes. (**A**) High abundance of *Fusobacterium* in HNSCC tumor tissues was positively associated with non-smokers, low T classification, better disease specific survival, and lower recurrence of disease. (**B**) Both univariate and multivariate regression analyses demonstrated that the high amount of *Fusobacterium* was an independent predictor of HNSCC disease specific survival. (**C**) A higher relative abundance of *Fusobacterium* in tumor tissue was correlated with improved 3-year disease-specific survival (DSS) and disease-free survival (DFS). (**D**) Quantitative PCR (qPCR) targeting the *nusG* gene confirmed the high abundance of *Fusobacterium nucleatum* in HNSCC tumor tissue.

In order to explore the association of the bacterial microbiome with patient outcomes, we compared the relative abundances of bacterial genera with patient factors, including age, gender, smoking, alcohol, T stage, N stage, relapse, and survival. Interestingly, *Fusobacterium* enrichment in HNSCC tumor tissues was significantly associated with non-smokers (mean abundance of 18.7% vs. 12.5%, *p* = 0.027), lower tumor stage (19.9% vs. 13.2%, *p* = 0.011), better cancer-specific survival (18.0% vs. 11.0%, *p* = 0.017), and a lower rate of relapse (18.5% vs. 11.0%, *p* = 0.008) (Figure 3A and Figure S4A). The association was further confirmed by pairwise comparison between tumor and the adjacent normal tissues, for example, in non-smokers (18.7% vs. 8.6%, *p* ≤ 0.001) but not in smokers (12.5% vs. 8.6%, *p* = 0.132). Using the third quartile of *Fusobacterium* relative abundance in the adjacent normal tissues as a cutoff point (12.3%), we divided the surveyed HNSCC patients into *Fusobacterium*-high (*n* = 38) and -low (*n* = 30) groups based on the abundance values of *Fusobacterium* in tumor tissues. Both univariate and multivariate regression analyses demonstrated that the abundance of *Fusobacterium* was an independent predictor of HNSCC-specific survival (Figure 3B), with significantly better 3-year disease-specific survival (DSS) (86.7% vs. 47.6% at 36 months, *p* ≤ 0.001) and disease-free survival (DFS) (85.0% vs. 41.8% at 36 months, *p* ≤ 0.001) (Figure 3C). Evaluation of 16Sr RNA gene short reads using a phylogenetic topology algorithm and qPCR validation targeting on the conserved *nusG* gene confirmed that these clinical outcomes are related to *F. nucleatum* (Figure 3D and Figure S4B,C). The effect of *F. nucleatum* enrichment in HNSCC tumor tissues was not significantly influenced by age, gender, alcohol consumption, or N stage.

We also observed several other bacteria associated with patient outcomes (Figure S5). For example, HNSCC tumor tissue-enriched *Catonella* and *Johnsonella* were usually observed in non-smokers or non-drinkers. The amount of *Peptostreptococcus* was positively related to more advanced tumor stage. Depletions of *Neisseria*, *Haemophilus*, and *Rothia* in HNSCC cases were associated with worse cancer-specific survival.

### 3.6. Fusobacterium nucleatum Localizes to the Intracellular Compartments of Tumor Cells

**Figure 4:**
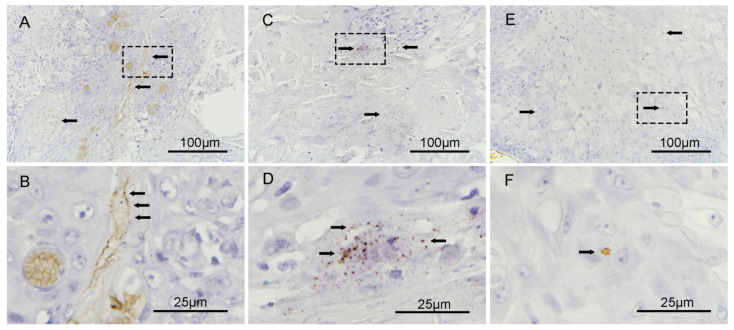
RNA ISH analysis of *F. nucleatum* in HNSCC tumors. (**A**) and (**B**) demonstrate an *F. nucleatum* biofilm-like lining on the surfaces of the tumor cells at 200×; the black arrows point to the biofilm. (**C**) and (**D**) demonstrate the intracellular localization of *F. nucleatum* in tumor cells located adjacent to keratin whorls of squamous cell carcinoma at 200×. (**E**) and (**F**) further demonstrate the localization of *F. nucleatum* in the intracellular compartments of tumor cells in an oral cavity squamous cell carcinoma at 200×.

*F. nucleatum* has been reported as a highly invasive pathogen. To determine the spatial location of *F. nucleatum* in HNSCC tumors, RNA in situ hybridization (ISH) analysis for *F. nucleatum* was performed on six qPCR positive tumors from the surveyed cohort. Invasive *F. nucleatum* was noted focally in isolated cells with a morphology consistent with malignant cells and in the biofilm of mucosa adjacent to the tumor (Figure 4). There was no *F. nucleatum* noted in the stroma.

### 3.7. Enrichment of Fusobacterium is Associated with Host Gene Promoter Methylation in HNSCC

To evaluate the potential effects of HNSCC-related bacteria in host genetic and epigenetic alterations, we performed CpG DNA methylation using bisulfite capture sequencing in a random subset of paired tumor tissues and adjacent normal tissues, including 18 patients grouped as *Fusobacterium*-high and 13 as *Fusobacterium*-low. We mainly focused on CpG islands in the promoter region (up to 3kb) because of its association with gene expression. Overall, 2503 and 7432 differentially methylated regions (DMRs) were hyper- and hypo-methylated in HNSCC tumors when compared to adjacent normal tissues (≥15% methylation difference, adjusted *p* ≤ 0.01) and may regulate 1316 and 4515 genes, respectively (Figure S6A and Table S5). Genes with conflicting DMRs were removed (*n* = 366). By integrating HNSCC tumor tissue RNA-seq transcriptome profiles from The Cancer Genome Atlas (TCGA) and an enrichment analysis using KEGG human database, 156 genes with hyper-methylated promoters in our surveyed tumor tissues also had downregulated gene expression in the TCGA HNSCC cohort (log2FC ≤ −1, adjusted *p* ≤ 0.01) and involved multiple pathways, including the cholinergic synapse (*CHRM2*, *GNG4*, *KCNJ12*, *ITPR1*, *PIK3R1*), AMPK signaling pathway (*CAB39L*, *LEPR*, *PIK3R1*, *CPT1B*, *CFTR*), peroxisome (*ACOX2*, *EPHX2*, *IDH2*, *ACOX3*), and PI3K–Akt signaling pathway (*CHRM2*, *RELN*, *GNG4*, *CHAD*, *FGF20*, *PIK3R1*, *EPHA2*, *FGF10*) (*p* ≤ 0.01) (Figure S6B). Similarly, 494 highly expressed genes in TCGA cohort (log2FC ≥ 1, adjusted *p* ≤ 0.01) were found to be hypo-methylated in their promoter regions, with the top five most enriched KEGG pathways being related to cytokine–cytokine receptor interaction, cell adhesion, osteoclast differentiation, amoebiasis, and ECM–receptor interaction (Figure S6C).

**Figure 5:**
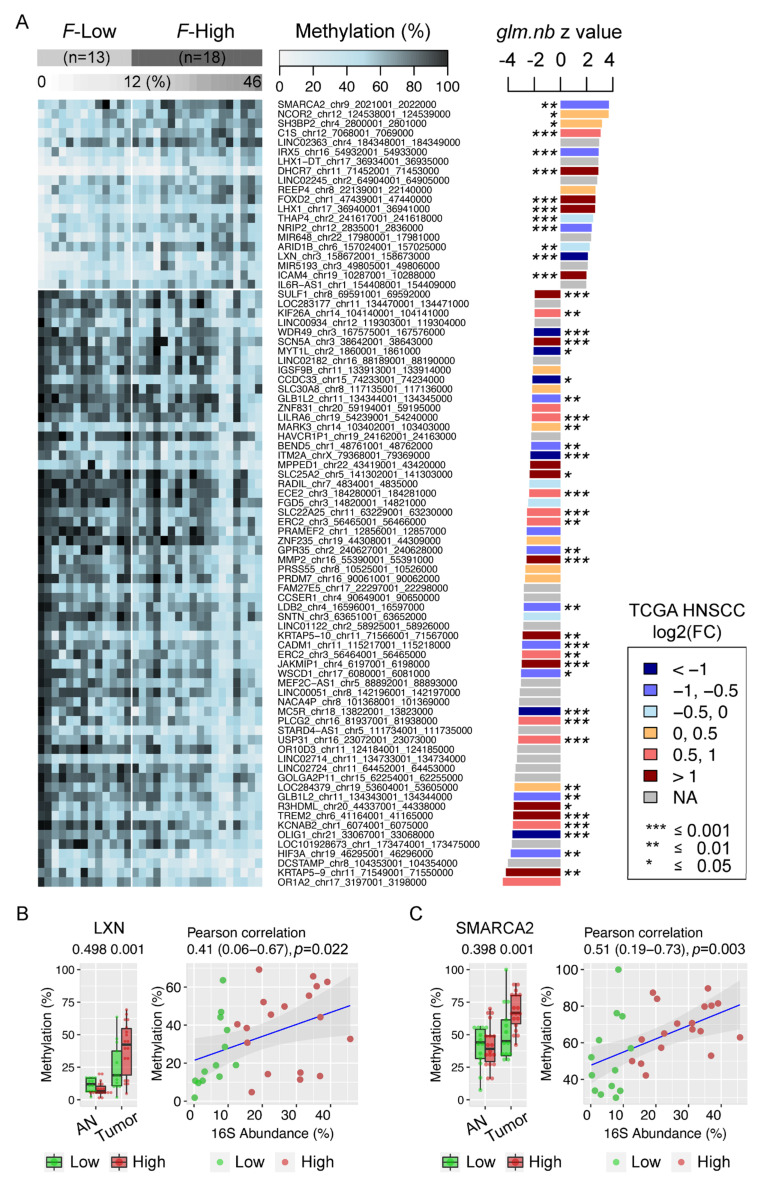
Bisulfite DNA capture sequencing demonstrating differentially methylated human gene promoters in *Fusobacterium*-enriched HNSCC tumor tissues. (**A**) A heat map of methylation profiles of host gene promoters between *Fusobacterium*-high (*F*-High) and *Fusobacterium*-low (*F*-Low) HNSCC tumor tissues. Negative binomial generalized linear regression model z values (*glm.nb*, *p* ≤ 0.05) are shown in the right panel of the heat map, with colors indicating gene expression changes (log2FC) in the TCGA HNSCC cohort. (**B**) Positive correlation between *Fusobacterium* enrichment and TSG-*LXN* promoter hypermethylation in HNSCC tumor tissues. (**C**) Positive correlation between *Fusobacterium* enrichment and TSG-*SMARCA2* promoter hypermethylation in HNSCC tumor tissues. AN: adjacent normal tissues. Tumor: HNSCC tumor tissues.

Next, we identified promotor regions that were differentially methylated between *Fusobacterium*-high and *Fusobacterium*-low tumor tissues to investigate whether HNSCC-enriched microbiota are associated with host gene methylation profiles. Interestingly, 20 (20) hyper- and 63 (61) hypo-methylated DMRs (genes) were positively and negatively related to *Fusobacterium* enrichment (≥15% methylation difference, adjusted *p* ≤ 0.01), respectively, which was further confirmed by a negative binomial generalized linear regression model test (*glm.nb*) and a Spearman’s rank-order correlation test (*p* ≤ 0.05) (Figure 5A and Figure S7, Table S5). We further compared gene expression profiles in the TCGA HNSCC cohort and found consistent associations between hypermethylation and downregulation in genes *LXN*, *IRX5*, *NRIP2*, and *SMARCA2* (log2FC ≤ −0.5, adjusted *p* ≤ 0.01). Tumor suppressor gene (TSG) latexin (*LXN*) expression negatively correlates with the prognosis of several human cancers and plays significant roles in inflammatory response, tumor cell migration, invasion and apoptosis (Figure 5B); and TSG-*SMARCA2* is involved in ATP-dependent chromatin remodeling related to DNA repair and replication (Figure 5C), implying a potential role of *F. nucleatum* infection in gene dysregulation related to the inflammatory response and cell proliferation through epigenetic silencing. Similarly, *F. nucleatum* may be also associated with promoted gene expression in HNSCC, including *SULF1*, *TREM2*, *MMP2*, *KRTAP5-10*, *SCN5A*, *JAKMIP1*, *KRTAP5-9*, *ECE2*, *LILRA6*, *KCNAB2*, *ERC2*, *KIF26A*, *USP31*, *PLCG2*, and *SLC22A25* (log2FC ≥ 0.5, adjusted *p* ≤ 0.01), given the observed hypomethylation of gene promoters related to the enrichment of *F. nucleatum*.

## 4. Discussion

In this study, we sought to characterize oral microbiota dysbiosis in a subgroup of HPV-negative HNSCCs and their interactions with clinical outcomes and host epigenetic alteration. Our findings revealed specific oral microbial signatures in HNSCC with significant enrichment of *Fusobacterium* and depletion of *Streptococcus*, which is consistent with other studies [1,15,17,19]. We observed a consistent bacterial pattern in both tissue and oral rinse communities, including twelve bacterial genera that provided microbial signatures in HNSCC with high sensitivity and specificity, thereby offering reliable applications as novel biomarkers to complement cancer diagnostics [20,21,22,23]. Network analysis found collaborative relationships between periodontal pathogens or between commensal taxa, implying distinct roles of oral bacteria in forming community in disease progresses.

We identified HNSCC non-smokers as having a higher abundance of *Fusobacterium nucleatum* in tumor tissues, while the enrichment in smokers was not significant. Associations of lower tumor stage and improved cancer-specific survival with enrichment of *F. nucleatum* in HNSCC cases were also novel observations. The finding of better clinical outcomes associated with elevated levels of *F. nucleatum* in HNSCC when comparing tumor with adjacent normal tissue is, however, in contrast to poorer survival and worse prognosis of *F. nucleatum* infection in colorectal cancer (CRC) [37,38,39] and esophageal cancer [40]. Long-term smoking, alcohol consumption, and high-risk human papillomavirus (HPV) infection are established oncogenic drivers in HNSCC [41]. For HNSCC patients lacking these traditional risk factors, however, chronic infection and inflammation as factors in carcinogenic feedback loops incorporating the resident microbiota are increasingly recognized in the pathogenesis of cancer, highlighting a potential role of *F. nucleatum* acting as a “driver” in premalignant and/or early lesions of HNSCC that may initiate cancer development, which is subsequently outcompeted by opportunistic bacterial passengers, resulting in a lower abundance at later stages of disease [42,43]. This hypothesis is in conjunction with known *F. nucleatum* pro-carcinogenic features associated with bacterial invasion and inflammation (e.g., flagella assembly and bacterial chemotaxis) [24,25,27], metabolic pathways (e.g., homolactic fermentation) [26], production of DNA-damage compounds [44], and promoted cell proliferation (e.g., E-cadherin/β-catenin signaling via its FadA adhesin) [45], suggesting that enrichment of *F. nucleatum* in early stages of tumorigenesis is an independent risk factor in patients lacking major risk factors for HNSCC.

Interestingly, an integrative analysis identified that the enrichment of *F. nucleatum* was associated with host gene promoter methylation, which further implies its pathophysiologic role in regulating transcriptome level involvement in pro-inflammatory and cell proliferation processes [9,10]. The tumor suppressor gene (TSG) latexin (*LXN*) has been downregulated in a number of human cancers, including lymphoma, gastric carcinoma, and thyroid carcinoma [37,38,39], and the TCGA HNSCC cohort, in congruence with its promoter hypermethylation in *Fusobcaterium*-high tumor tissues. *LXN* has also been shown to be involved in the inflammatory response with inhibition of tumor growth and colony formation [40,42]. Overexpression of *LXN* inhibits the activity of the nuclear factor NF-kB pathway in inflammation by binding to a ribosomal protein subunit 3 (Rps3) [43], and also increased apoptosis and lowered proliferative activity [46]. This is in broad agreement with our observation of loss of *LXN* expression likely by *F. nucleatum*-associated epigenetic silencing in the HNSCC tumor microenvironment, where bacterial invasion may interfere with the pro-inflammatory immunomodulatory gene signature of *LXN* in blocking tumor immune cell evasion. We also observed a similar association between *F. nucleatum* enrichment and TSG-*SMARCA2* promoter hypermethylation. The genes encoding the ATPase of the chromatin remodeling SW1/SNF complexes *SMARCA4* and the alternative *SMARCA2* are usually mutated or silenced in tumors, suggesting a role as tumor suppressor [47]. The SWI/SNF complex has roles in regulating gene expression and cancer development. Mutations of *SMARCA2* are rarely observed but gene silencing related to epigenetic regulation in tumor has been reported [48,49], which is in line with strong hypermethylation observed in our *Fusobacterium*-enriched HNSCC tumor tissues when compared to *Fusobacterium*-low tumor tissues. In contrast to hypermethylation, hypomethylation of sulfatase 1 (*SULF1*) and matrix metalloproteinase 2 (*MMP2*) promoters was observed to be associated with the enrichment of *F. nucleatum* in our HNSCC tumor tissues. Both genes were overexpressed in human cancers, such as gastric, lung, or colorectal cancer [50,51], and were reported to remodel or breakdown extracellular matrix (ECM) [44], resulting in dysregulated cell signaling pathways (e.g., Wnt signaling pathway) [41], or release of growth factors such as TGF-β [45] that affect cell immune tolerance, adhesion, proliferation, and migration [52]. Whether *F. nucleatum* directly up-regulates *SULF1* and/or *MMP2* through epigenetic mechanisms or ECM remodeling by gene degradation plays a role in initial HNSCC tumor growth remains an interesting question warranting further investigations.

In this study, notable similarities in the relative abundances of bacteria were observed across both tissues and oral rinses, suggesting the potential to use oral rinses as non-invasive samples to reflect tissue microbial composition. There can be certain differences if difference sample courses are used, highlighting the importance of assessing the microbiota across both tissue and oral rinses, strengthening our findings. Given the prominent role of somatic mutations in TP53 in smoking-related HNSCCs, for example, dramatic enrichment of *F. nucleatum* in nonsmokers, may explain it in part as a pathogen causing DNA damage in patients without traditional high-risk factors [47,48,49]. However further prospective evaluations of patient factors including comorbidities will be needed to validate the clinical outcomes in *F nucleatum*-enriched HNSCC tumors. Furthermore, *F. nucleatum* may regulate host gene transcriptome profiles through epigenetic mechanisms and act as a pro-inflammatory driver involving multiple cell signaling pathways.

## 5. Conclusions

In conclusion, this study improves our understanding of microbial communities associated with HNSCC. Enriched *F. nucleatum* abundance in HNSCC non-smokers and an improved survival suggest possible pathologic roles of bacterial genera in patients that may serve as targets for HNSCC intervention through host epigenetic modifications. Future studies investigating the functional roles of HNSCC-related bacterial taxa in the host genomic and epigenetic changes will be essential to elucidating driver or passenger roles in HNSCC. Finally, a unique HNSCC microbial signature may facilitate the use of oral bacteria as biomarkers for disease screening and prevention.

## Figures and Tables

**Table 1:** Bacterial genera with coherent changes of the relative abundance between HNSCCs and controls across both tissues and oral rinses.

	AN	Tumor	Healthy	HNSCC	Tumor Tissue Compared to AN					HNSCC Oral Rinse Compared to Healthy Subjects				
Taxonomy (Phylum; Genus)	Abundance (Mean ± smd)	Abundance (Mean ± smd)	Abundance (Mean ± smd)	Abundance (Mean ± smd)	Difference in Abundance	*p* Value (mwu Test)	LEfSe assign	LEfSe *p* adj	AUC Value	Difference in Abundance	*p* value (mwu test)	LEfSe Assign	LEfSe *p* adj	AUC Value
Fusobacteria; Fusobacterium	8.60 ± 0.87	16.25 ± 1.51	4.60 ± 0.36	7.69 ± 0.72	7.65 (4.14,11.15)	0.0000	Tumor	0.0002	0.69 (0.60, 0.78)	3.09 (−0.42, 6.60)	0.0027	HNSCC	0.0025	0.65 (0.55, 0.74)
Firmicutes; Peptostreptococcus	0.79 ± 0.19	1.33 ± 0.19	0.06 ± 0.01	0.22 ± 0.06	0.54 (0.03, 1.05)	0.0042	Tumor	0.0095	0.63 (0.53, 0.72)	0.16 (−0.35, 0.67)	0.0132	HNSCC	0.0130	0.62 (0.52, 0.72)
Firmicutes; Streptococcus	17.16 ± 1.71	8.00 ± 1.33	15.00 ± 0.92	10.64 ± 1.02	−9.17 (−13.86, −4.48)	0.0000	AN	0.0000	0.75 (0.67, 0.83)	−4.36 (−9.05, 0.33)	0.0001	Healthy	0.0001	0.70 (0.61, 0.79)
Proteobacteria; Neisseria	6.80 ± 1.12	4.07 ± 0.96	18.82 ± 1.51	14.10 ± 1.28	−2.73 (−7.25, 1.79)	0.0076	AN	0.0400	0.60 (0.51, 0.70)	−4.72 (−9.24, −0.21)	0.0222	Healthy	0.0228	0.61 (0.52, 0.71)
Actinobacteria; Rothia	2.99 ± 0.48	1.21 ± 0.37	4.47 ± 0.57	1.77 ± 0.28	−1.78 (−3.39, −0.18)	0.0000	AN	0.0003	0.68 (0.59, 0.77)	−2.70 (−4.31, −1.10)	0.0000	Healthy	0.0000	0.75 (0.67, 0.83)
Actinobacteria; Actinomyces	1.99 ± 0.39	0.57 ± 0.16	0.87 ± 0.11	0.69 ± 0.13	−1.42 (−2.24, −0.60)	0.0000	AN	0.0000	0.73 (0.65, 0.82)	−0.18 (−1.00, 0.64)	0.0100	Healthy	0.0097	0.63 (0.53, 0.72)
Firmicutes; Granulicatella	1.42 ± 0.20	0.91 ± 0.25	1.20 ± 0.14	1.10 ± 0.40	−0.52 (−1.48, 0.45)	0.0126	AN	0.0127	0.62 (0.53, 0.72)	−0.10 (−1.07, 0.87)	0.0002	Healthy	0.0002	0.68 (0.59, 0.77)
Proteobacteria; Lautropia	0.47 ± 0.14	0.05 ± 0.02	0.64 ± 0.14	0.27 ± 0.07	−0.42 (−0.80, −0.04)	0.0008	AN	0.0096	0.60 (0.53, 0.67)	−0.37 (−0.75, 0.00)	0.0000	Healthy	0.0000	0.71 (0.62, 0.80)
Proteobacteria; Cardiobacterium	0.13 ± 0.04	0.05 ± 0.02	0.09 ± 0.01	0.09 ± 0.03	−0.09 ( −0.18, 0.01)	0.0192	AN	0.0410	0.58 (0.50, 0.65)	−0.00 (−0.10, 0.10)	0.0020	Healthy	0.0020	0.65 (0.56, 0.73)
Actinobacteria; Corynebacterium	0.15 ± 0.04	0.08 ± 0.05	0.27 ± 0.03	0.09 ± 0.03	−0.07 (−0.21, 0.08)	0.0008	AN	0.0091	0.60 (0.53, 0.68)	−0.18 (−0.32, −0.04)	0.0000	Healthy	0.0000	0.80 (0.72, 0.87)
Firmicutes; Abiotrophia	0.14 ± 0.08	0.08 ± 0.04	0.08 ± 0.02	0.02 ± 0.01	−0.06 (−0.22, 0.11)	0.1402	AN	0.0106	0.58 (0.52, 0.65)	−0.06 (−0.23, 0.10)	0.0001	Healthy	0.0001	0.66 (0.59, 0.73)
Firmicutes; Oribacterium	0.77 ± 0.16	0.72 ± 0.25	0.15 ± 0.02	0.11 ± 0.02	−0.05 (−0.59, 0.49)	0.0383	AN	0.0050	0.64 (0.54, 0.73)	−0.04 (−0.58, 0.49)	0.0123	Healthy	0.0121	0.62 (0.53, 0.71)

## Supplementary Materials

The following are available online at https://www.mdpi.com/2072-6694/12/11/3425/s1. Figure S1: A refinement of twelve HNSCC-related bacterial genera discriminating HNSCCs from controls both in tissues (tumor vs adjacent normal) and oral rinses (HNSCC vs healthy). Figure S2: Bacterial genera showing different preference of colonization between tissue and oral rinse communities. Figure S3: Microbial Functional prediction based on 16S rRNA gene community composition using PICRUSt2. Figure S4: Association of Fusobacterium abundance with HNSCC patient outcomes. Figure S5: Association of other bacterial genera abundance with HNSCC patient outcomes. Figure S6: Bisulfite DNA capture sequencing of paired tumor and AN tissue samples from thirty-one HNSCC patients. Figure S7: Heat map of methylation profiles of host gene promoters showing differentially methylated status between Fusobacterium-high and Fusobacterium-low HNSCC tumor tissues. Table S1: Demographic information of patients with HNSCC compared to the healthy subjects. Table S2: Relative abundance of bacterial genera in designated groups. Pairwise Tukey HSD post-hoc test and non-parametric Wilcoxon Signed Rank test were used to detect statistical significance. Table S3: Discriminant analysis of predicted KEGG KO pathways between cases and controls across tissues and oral rinses using DESeq2. Table S4: Contribution in relative abundance of oral bacteria in predicted KEFF KO pathways. Table S5: Association of Fusobacterium enrichment with host gene promoter methylation.

## Abbreviations

HNSCC	Head and neck squamous cell carcinoma
AUC	Area under the receiver operating curve
HPV	Human papillomavirus
AN	Adjacent normal
ORF	Open reading frame
OUT	Operational taxonomic unit
ASV	amplicon sequence variant
PERMANOVA	Permutational multivariate analysis of variance
MWU	Mann–Whitney Wilcoxon rank sum test
WSR	Wilcoxon signed rank test
KW	Kruskal–Wallis test
Tukey HSD	Tukey’s honest significant difference
ROC	Receiver operating characteristic
KEGG	Kyoto Encyclopedia of Genes and Genomes
KO	Kyoto Encyclopedia of Genes and Genomes Orthology
FFPE	Formalin-fixed paraffin-embedded
DMRs	Differentially methylated regions
TCGA	The cancer genome atlas
HR	High risk
PCoA	Principal coordinate analysis
LefSe	Linear discriminant analysis effect size
LDA	Linear discriminant analysis
PICRUSt2	Phylogenetic Investigation of Communities by Reconstruction of Unobserved States 2
DSS	Disease specific survival
DFS	Disease free survival
ISH	In situ hybridization
Glm nb	generalized linear regression model test
TSG	tumor suppressor gene
LXN	Latexin
qPCR	Quantitative PCR
F-Low	Fusobacterium-low
F-High	Fusobacterium-high
