# Supplementary material for: The Intersection between Oral Microbiota, Host Gene Methylation and Patient Outcomes in Head and Neck Squamous Cell Carcinoma
Source: Cancers (Basel). 2020 Nov 18;12(11):3425. doi: 10.3390/cancers12113425 (PMC7698865; doi:10.3390/cancers12113425)
Supplement: Supplementary file 1 [file cancers-12-03425-s001.zip › cancers-982442-supplement.docx]

Supplementary Materials

The Intersection between Oral Microbiota, Host Gene Methylation and Patient Outcomes in Head and Neck Squamous Cell Carcinoma

Zigui Chen, Po Yee Wong, Cherrie W. K. Ng, Linlin Lan, Sherwood Fung, Jing-Woei Li, Liuyang Cai, Pu Lei, Qianqian Mou, Sunny H. Wong, William K. K. Wu,Ryan J. Li, Katie Meehan, Vivian W. Y. Lui, C Chow, Kwok-Wai Lo, Amy B. W. Chan, Siaw Shi Boon, Eric H. L. Lau, Zenon Yeung, K. C. Allen Chan, Eddy W. Y. Wong, Alfred Sze-Lok Cheng, Jun Yu, Paul K. S. Chan and Jason Y. K. Chan


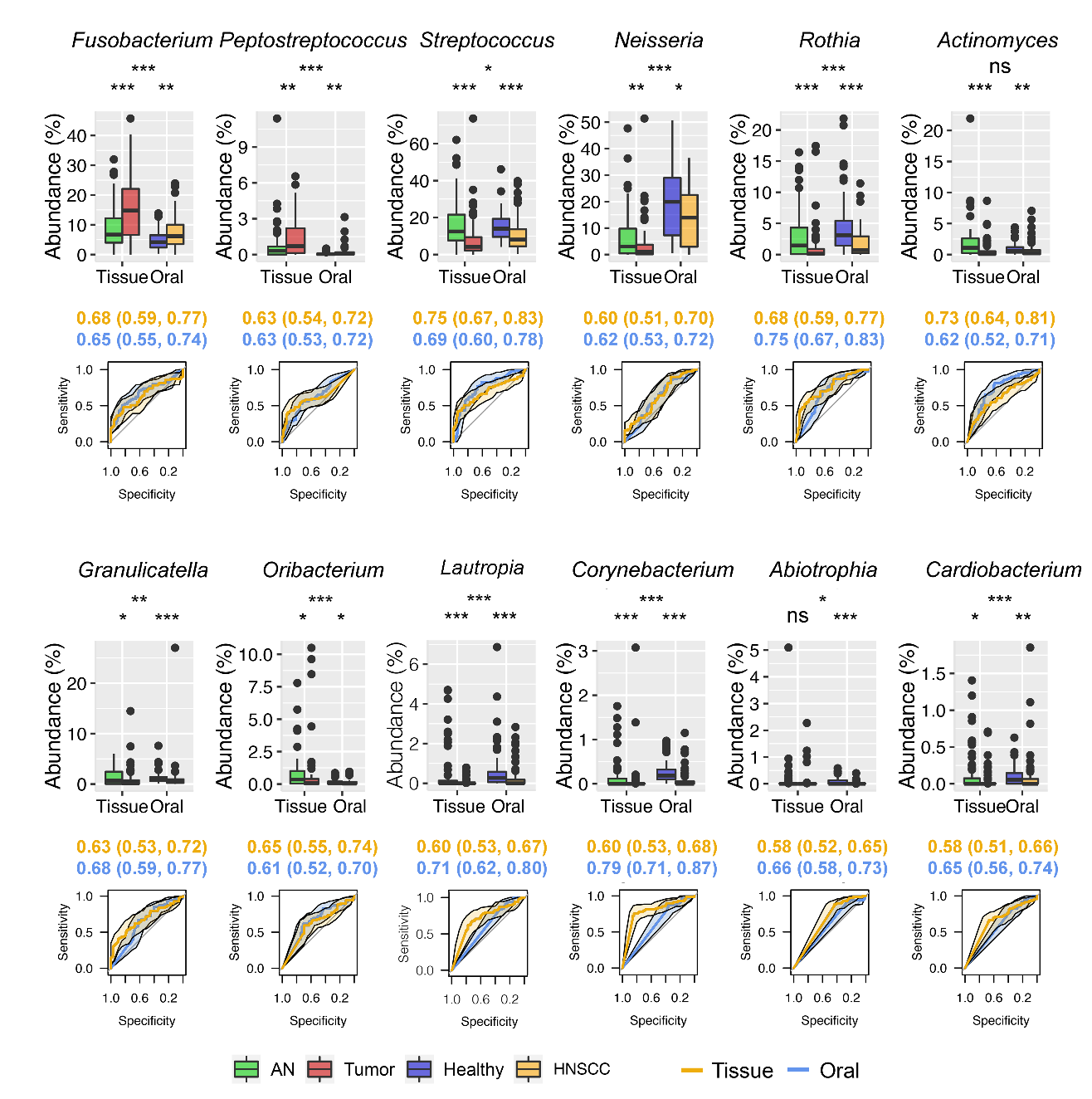


Figure S1: A refinement of twelve HNSCC-related bacterial genera discriminating HNSCCs from controls both in tissues (tumor vs adjacent normal) and oral rinses (HNSCC vs healthy).


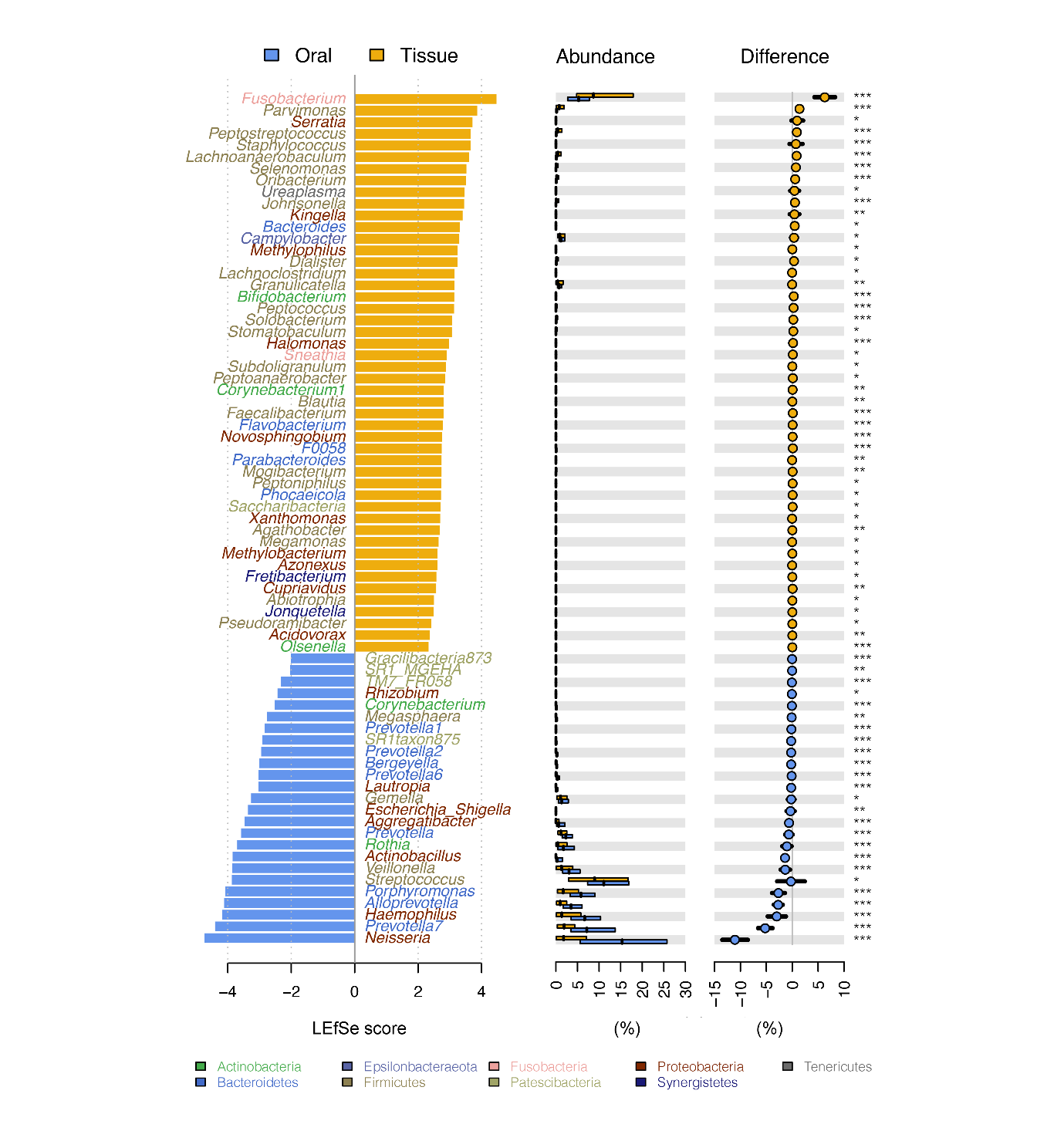


Figure S2: Bacterial genera showing different preference of colonization between tissue and oral rinse communities.


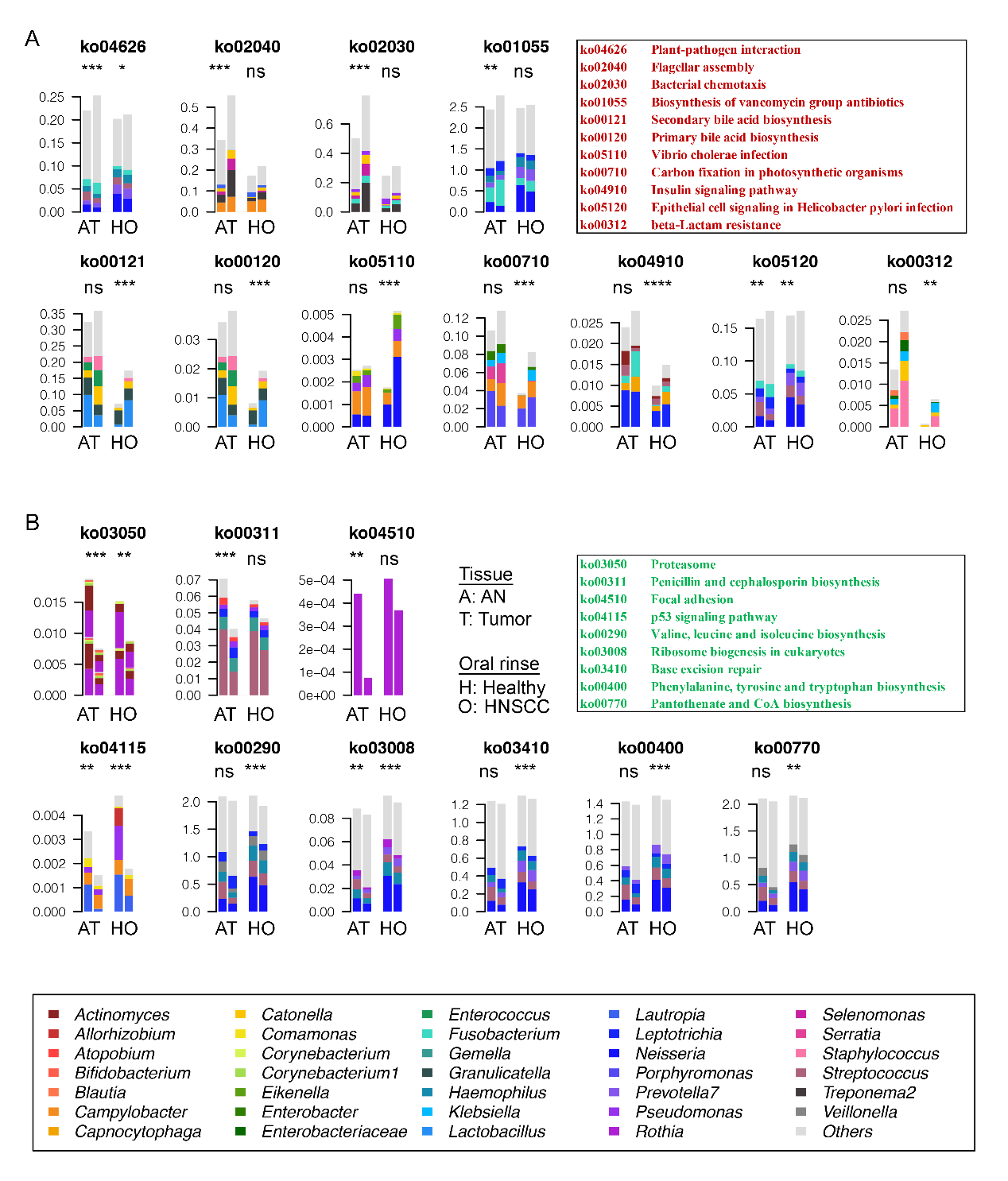


Figure S3: Microbial Functional prediction based on 16S rRNA gene community composition using PICRUSt2.


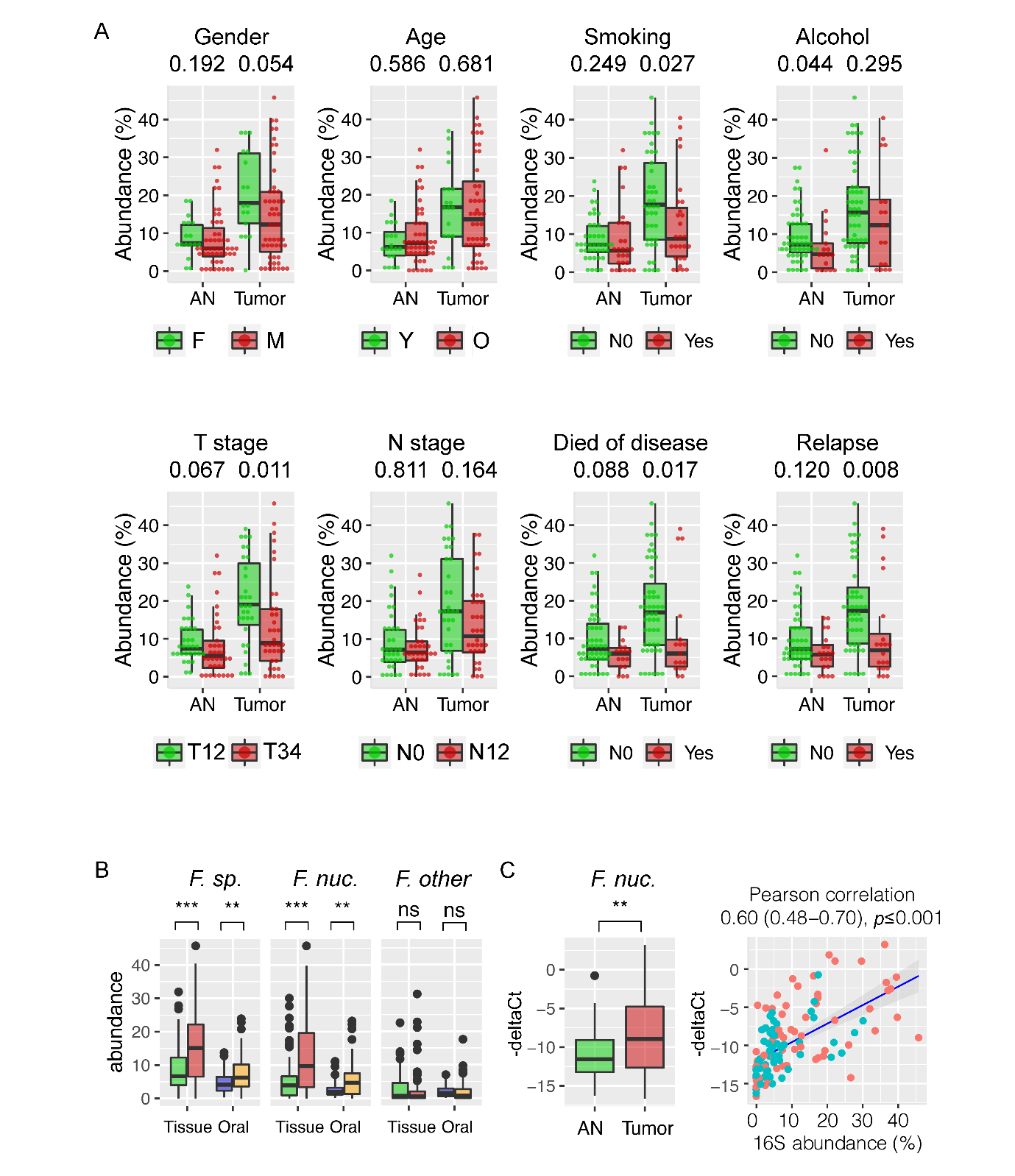


Figure S4: Association of Fusobacterium abundance with HNSCC patient outcomes.


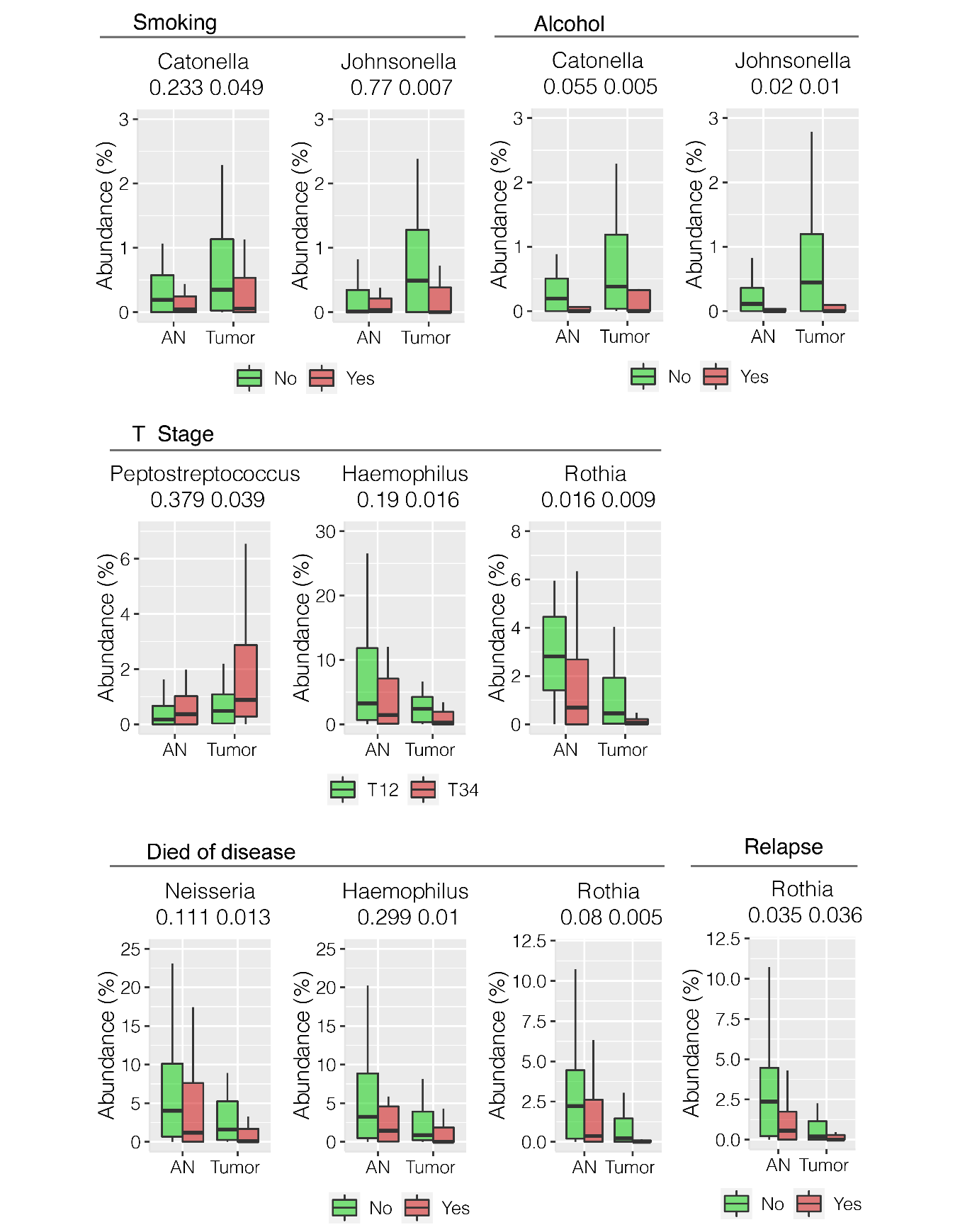


Figure S5: Association of other bacterial genera abundance with HNSCC patient outcomes.


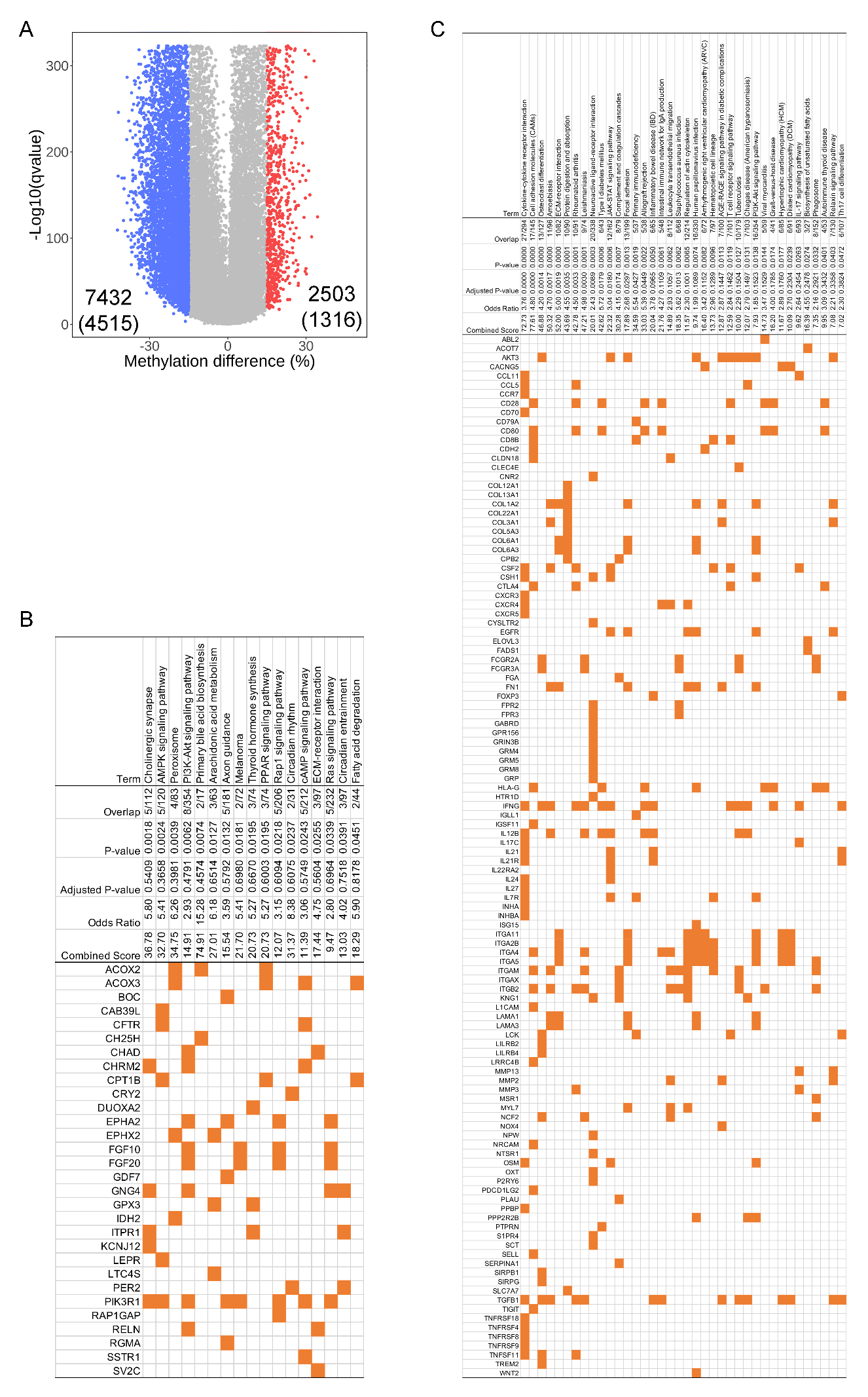


Figure S6: Bisulfite DNA capture sequencing of paired tumor and AN tissue samples from thirty-one HNSCC patients.


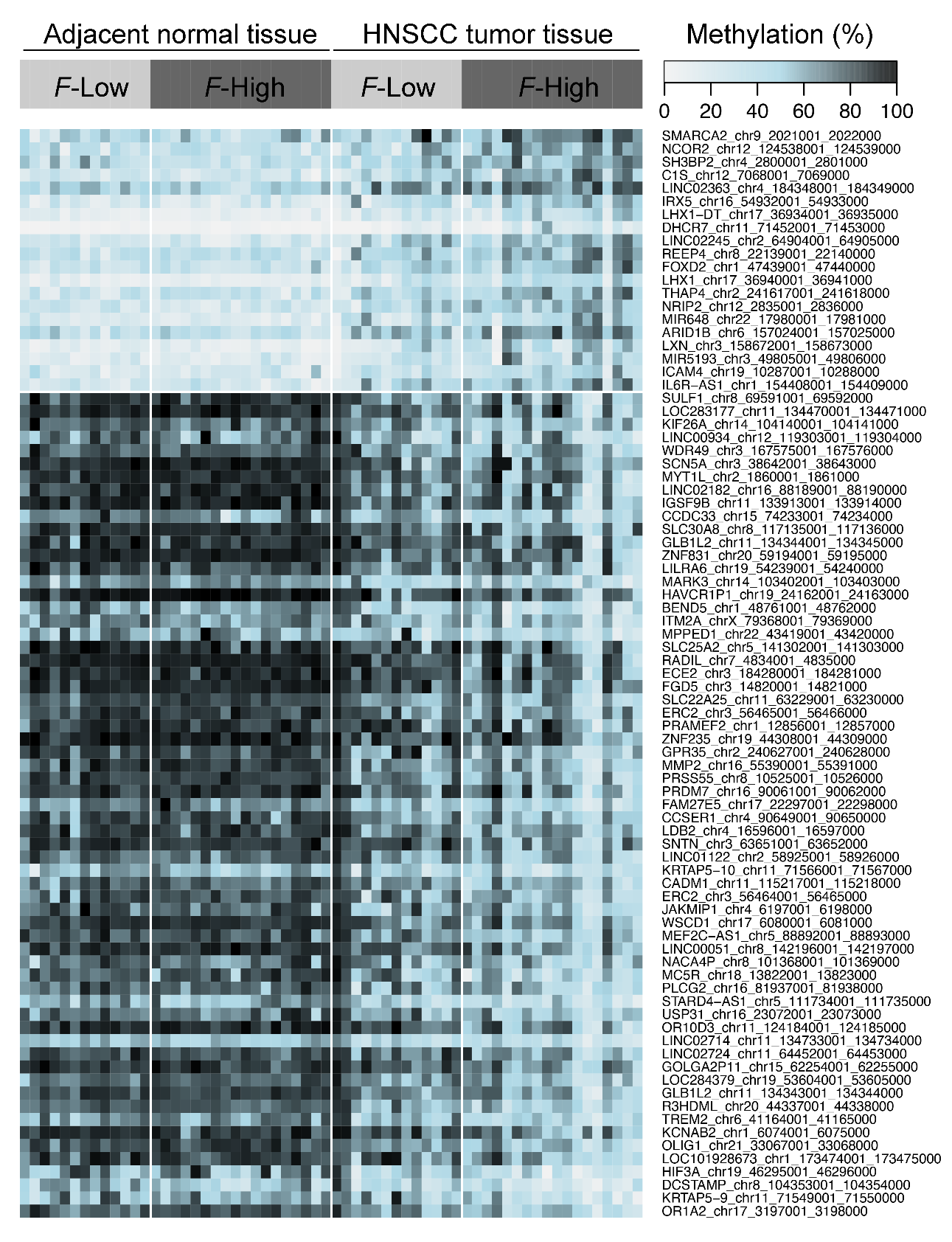


Figure S7: Heat map of methylation profiles of host gene promoters showing differentially methylated status between Fusobacterium-high and Fusobacterium-low HNSCC tumor tissues.
